# Understanding emotional influences on sustained attention: a study using virtual reality and neurophysiological monitoring

**DOI:** 10.3389/fnhum.2024.1467403

**Published:** 2024-10-17

**Authors:** Yang Shen, Huijia Zheng, Yu Li, Xuetao Tian

**Affiliations:** ^1^China Basic Education Quality Monitoring Collaborative Innovation Center, Beijing Normal University, Beijing, China; ^2^Peabody College of Education and Human Development, Vanderbilt University, Nashville, TN, United States; ^3^Beijing Key Laboratory of Applied Experimental Psychology, National Demonstration Center for Experimental Psychology Education, Faculty of Psychology, Beijing Normal University, Beijing, China

**Keywords:** sustained attention, emotion, virtual reality, electroencephalogram, photoplethysmography

## Abstract

**Introduction:**

Emotion and attention regulation significantly influence various aspects of human functioning and behavior. However, the interaction between emotion and attention in affecting performance remains underexplored. This study aims to investigate how individual differences in sustained attention, influenced by varying emotional states.

**Methods:**

A total of 12 participants underwent emotion induction through Virtual Reality (VR) videos; completed an AX-CPT (continuous performance test) task to measure sustained attention, for which task performance is evaluated from two aspects, task accuracy and task reaction times; and reported their flow states. EEG and PPG data were collected throughout the sessions, as supporting evidence for sustained attention.

**Results:**

Our findings suggest that emotional valence and arousal significantly influence task reaction times and sustained attention, when gender differences are accounted for, but do not significantly impact task accuracy. Specifically, males responded faster under high-arousal negative emotions, while females responded faster under high-arousal positive emotions. Additionally, we find that flow experience is not significantly impacted by emotions states or sustained attention.

**Discussion:**

The study underscores the nuanced interplay between emotion, sustained attention, and task performance, suggesting that emotional states can differentially impact cognitive processes. Also, it support the use of VR, EEG, and PPG technologies in future research on related topics. Future research could expand upon this study by including larger sample sizes and a wider range of emotional inductions to generalize the findings.

## 1 Introduction

Emotion and attention regulation both play important roles in adaptive functioning and behavior (Pekrun et al., 2002; Westphal et al., 2018; Whitehill et al., 2014). In daily life, we are constantly faced by large amounts of information, which we use attention to filter and process (Brosch et al., 2013); emotions, in turn, pose effect upon our attention profiles, changing the portion of information that we attend to, thus modulating behavior (Mitchell, 2022). Recently, the interaction between emotion and attention have come into focus in the fields of education and psychology (Wass et al., 2021; Baykal, 2022), as the specific process in which they impact performance remains unclear. In related research, a commonly applied theory of emotion is the dimensional theory, which distinguishes emotions based on their positions in a continuous multi-dimensional space, characterized by two primary dimensions: valence (degree of positivity or negativity of the emotion) and arousal (physiological activation of the emotion, high to low) (Russell, 1980). Existing research have shown that emotional arousal and valence can modulate attention allocation and selection (Compton, 2003; Phelps et al., 2006). However, findings on the specific relationship between different emotional dimensions and attention have yet to concur. Some studies indicate that high-arousal emotions increase attention directed to high-priority stimuli, but decrease attention toward low-priority stimuli (Mather and Sutherland, 2011). Other studies have identified that negative emotions can lead to global attention, whereas positive emotions lead to more local attention (Gasper and Clore, 2002). Further complicating the picture, arousal and valence also interact to produce varied effects on task performance, possibly related to their moderation on attention. For instance, research found that low-arousal negative affect enhances target recognition accuracy, high-arousal negative affect lower target accuracy, while positive affect’s influence on target accuracy do not differ significantly with different level of arousal (Jefferies et al., 2008).

Hence, it seems necessary to unify these research outcomes into a more comprehensive picture. In this study, we use task-specific sustained attention, i.e., the ability to maintain focus on the experiment task, as a bridging factor between attention and task performance, while also taking individual differences into consideration, in hopes for explicating the influence of different emotional dimensions on attention and task performance. This is measured through reaction time and accuracy performance on an AX-CPT (continuous performance test) task. In order to further clarify the picture, we also take one step forward from existing research by employing new methodologies to improve validity. Immersive Virtual Reality (VR) technology is used to enhance the ecological validity of emotion induction, in comparison to traditional induction measures, while sustained attention is additionally assessed through objective measures including electroencephalogram (EEG) and photoplethysmography (PPG), which could likely reflect sustained attention more directly than common subjective/indirect measures. To this end, we also integrate prior findings on physiological response to task activation, to form a set of signals that could be representative of sustained attention. All in all, our primary goal for this study is to explore how different valence and arousal of emotions impact sustained attention, as demonstrated by EEG and PPG data. Along the way, we also validate the application of VR, EEG and PPG technology in future related studies.

In line with this, we test a secondary hypothesis as well. The flow state is a subjective experience of effortless concentration (Csikszentmihalyi, 1975). Research on its relationship with attention has yielded inconsistent results: some studies suggested that people who frequently experience the flow state show more sustained attention (Swann et al., 2012), while others found no significant relationship between flow and sustained attention, since flow is an automatic, unconscious process, while sustained attention requires effort (Marty-Dugas and Smilek, 2019; Schiefele and Raabe, 2011; Ullen et al., 2012). These conflicts bring up our secondary hypothesis: Is experiencing the flow state related to sustained attention and emotion valence/arousal? If participants tend to experience flow states more frequently during certain attention/emotion states, this may also lead to differential task performance, and thus produce confused results (Harris et al., 2021). This hypothesis serves to resolve one more factor that may confound the effect of different emotion states on attention and task performance.

## 2 Materials and methods

### 2.1 Stimuli, paradigms and equipment

This study aims to investigate the impact of different emotional states on sustained attention, with a side focus on flow. The necessary instruments are detailed below.

**FIGURE 1:**
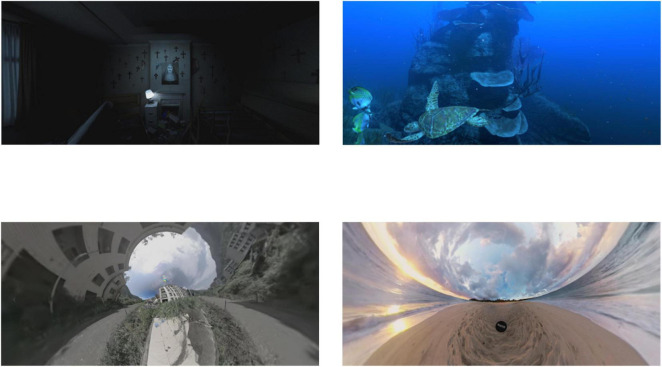
Screenshots of four VR videos used for emotion induction, including “negative-valence, high-arousal,” “positive-valence, high-arousal,” “negative-valence, low-arousal,” and “positive-valence, low-arousal.” Images reproduced from Li et al. (2017).

Immersive VR videos were utilized to induce different emotional states, presented in a HTC Vive Pro HMD. These were selected from the Stanford Immersive Virtual Reality Video Database (Li et al., 2017), which contains 73 VR clips categorized according to emotion valence and arousal (Russell, 1980). In the end, four videos with scores closest to the quadrant extremes of the valence and arousal dimensions were chosen to achieve optimal emotion induction, as shown in Figure 1.

A subjective emotion self-report scale, the Self-Assessment Manikin (SAM; Bradley and Lang, 1994), was used to evaluate participants’ emotion arousal and valence. The AX-CPT (continuous performance test) paradigm was used to assess sustained attention. In AX-CPT, participants are instructed to respond to letter sequences, with “X” as the target stimulus, but only if preceded by the letter “A”. The sequences include four types: AX (target), AY, BX, and BY, where “B” can be any letter other than “A,” and “Y” can be any letter other than “X.” The classic AX-CPT paradigm comprised 70% AX sequences and 10% each of AY, BX, and BY sequences (Braver et al., 2007). Participants’ accuracy and reaction times were recorded during the task as performance criteria. The Flow Short Scale (Engeser and Rheinberg, 2008), a 10-item Likert scale, was used to measure flow experience during the AX-CPT task.

**FIGURE 2:**
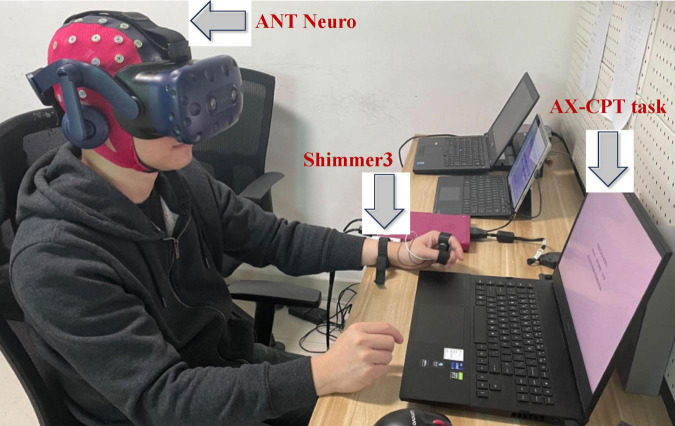
Experimental equipment.

During the AX-CPT task, a Shimmer3 wearable device was used for ECG data collection, and an ANT Neuro system was used for EEG data collection, as shown in Figure 2.

### 2.2 Participants

A total of twelve participants were recruited for this study, 6 male and 6 female, aged 21 to 45 years (mean age = 31.2), right-handed and with normal or corrected-to-normal vision. Fields of expertise of the participants include computer science, education, psychology, foreign languages, marketing, and applied chemistry. All participants had no prior experience with similar experiments.

### 2.3 Procedure

**FIGURE 3:**
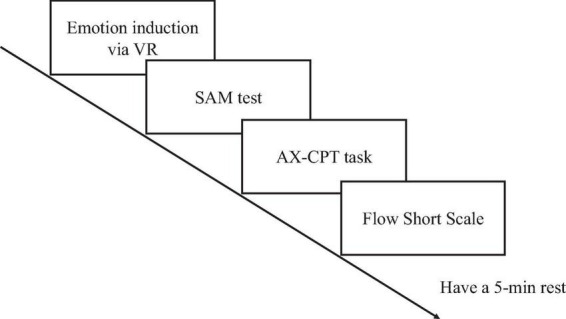
The steps in one session of the experiment.

The experiment was conducted one participant at a time without time limit, guided by a trained researcher in a quiet room and under constant screen brightness. Before the experiment, participants were introduced to the study, asked to sign a consent form, prepared for EEG and PPG collection, and instructed to fill out a demographic questionnaire covering gender, age, occupation, handedness, as well as previous VR experience. Then, participants were asked to stay stationary for 5 min, where a baseline SAM score was acquired. The experiment consists of four sessions, each with five steps, as shown in Figure 3. First, participants were randomly assigned to one of four conditions, each representing one of the four emotional dimensions: negative-valence high-arousal, negative-valence low-arousal, positive-valence high-arousal, or positive-valence low-arousal, to receive corresponding emotion induction through a VR video approximately 3 min and 30 s long. Afterwards, participants filled out the SAM scale, completed the AX-CPT attention task, and filled out the Flow Short Scale. Finally, participants take a 5-min rest before the next session.

### 2.4 Electroencephalogram (EEG) pre-processing

Before EEG data analysis, preprocessing was performed with EEGLAB (Delorme and Makeig, 2004), an open-source toolbox based on MATLAB, during which a band-pass FIR filter from 3 Hz to 47 Hz was applied.

### 2.5 Photoplethysmography (PPG) data

This study analyzed Heart Rate Variability (HRV), as calculated from PPG, to reflect autonomic responses that result from the sustained attention task. HRV analysis generally consists of two aspects, time-domain features and frequency-domain features.

Time-domain features reflect heart rate variability and autonomic regulation, including:

• MEANRR: Mean reciprocal of RR intervals, indicating heart rate stability, where RR interval is the time interval between two peaks.
• MEDIANRR: Median RR intervals, providing a robust measure against outliers.
• MEANHR: Mean heart rate, linked to cardiovascular health and autonomic function.
• SDNN: Standard deviation of NN intervals, reflecting overall HRV, where NN interval is the normal heartbeat interval.
• RMSSD: Root mean square of successive differences, indicating parasympathetic activity.
• pNN50: Percentage of NN intervals with differences over 50 ms, reflecting parasympathetic activity.

Frequency-domain features, which primarily reflects parasympathetic activity (Kumar et al., 2023), were analyzed using resampling, interpolation, and Fast Fourier Transform (FFT), to obtain Power Spectral Density (PSD). The final features includes:

• VLF: Very low frequency (0.0033–0.04 Hz), associated with long-term regulatory mechanisms.
• LF: Low frequency (0.04–0.15 Hz), reflecting sympathetic and parasympathetic activity.
• HF: High frequency (0.15–0.4 Hz), indicating parasympathetic (vagal) regulation.
• LF/HF Ratio: Evaluation on the balance between sympathetic and parasympathetic activity.
• Normalized LF (LF[n.u.]) and HF (HF[n.u.]): Relative power in their respective ranges, providing comparative importance via transforming absolute power to normalized units.

Absolute Power VLF (VLF[abs]), LF (LF[abs]), and HF (HF[abs]): Reflect the energy distribution in their respective frequency ranges, related to specific physiological mechanisms.

## 3 Results

### 3.1 Emotion induction

Due to the small sample size, we choose a non-parametric test, the Wilcoxon signed-rank test, to analyze differences in accuracy and reaction times in the AX-CPT task under different emotional states. Mean SAM scores among 12 participants are calculated for the four virtual reality videos. Firstly, we check to ensure that the emotion induction took effect. Results are presented in Table 1. Wilcoxon signed-rank tests showed significant differences in valence (*Z* = 3.07, *p* = 0.002) and arousal scores (*Z* = 3.01, *p* = 0.003) between corresponding videos. The virtual reality videos effectively elicited corresponding emotional responses.

**TABLE 1:** Valence and arousal scores from SAM scale.

Emotion dimension	Valence (M ± SD)	Arousal (M ± SD)
Negative-valence, low-arousal	3.17 ± 1.80	4.17 ± 2.17
Negative-valence, high-arousal	2.25 ± 1.66	8.75 ± 1.29
Positive-valence, high-arousal	7.08 ± 1.62	6.42 ± 1.83
Positive-valence, low-arousal	7.42 ± 1.08	5.00 ± 2.17

### 3.2 Emotion and AX-CPT task data

Using the Wilcoxon signed-rank test, we analyze accuracy rates and reaction times during the AX-CPT task under the four emotional dimensions, and consider the influence of gender on the results.

As reported in Table 2, accuracy rates for positive emotions (*M* = 98.33, *SD* = 1.13, *Mdn* = 98.25) and negative emotions (*M* = 98.50, *SD* = 1.19, *Mdn* = 98.75) show no significant difference (*Z* = 0.49, *p* = 0.620). Accuracy rates for high-arousal emotions (*M* = 98.29, *SD* = 1.01, *Mdn* = 98.50) and low-arousal emotions (*M* = 98.54, *SD* = 1.16, *Mdn* = 98.75) also show no significant difference (*Z* = 1.08, *p* = 10.280). Also, reaction times for positive emotions (*M* = 30098.58, *SD* = 5758.64, *Mdn* = 29501.00) and negative emotions (*M* = 28465.17, *SD* = 5166.93, *Mdn* = 28273.00) show no significant difference (*Z* = 1.73, *p* = 0.084). Similarly, reaction times for high-arousal (*M* = 29061.67, *SD* = 4897.09, *Mdn* = 29341.00) and low-arousal emotions (*M* = 29502.08, *SD* = 5712.97, *Mdn* = 28618.00) show no significant difference (*Z* = 0.784, *p* = 0.433).

**TABLE 2:** Influence of emotion valence and arousal on AX-CPT task performance.

Emotion dimension	Reaction time (M ± SD)	Accuracy (M ± SD)
Positive-valence	30098.58 ± 5758.64	98.33 ± 1.14
Negative-valence	28465.17 ± 5166.93	98.50 ± 1.19
High-arousal	29061.67 ± 4897.09	98.29 ± 1.01
Low-arousal	29502.08 ± 5712.97	98.54 ± 1.16

Further, as shown in Table 3, analyzing the influence of emotional valence, arousal, and gender on accuracy using the Wilcoxon signed-rank test revealed no significant differences (*Z* = 0.68, *p* = 0.500) in accuracy rates between males under high-arousal negative (*M* = 97.67, *SD* = 1.63, *Mdn* = 97.50) and high-arousal positive emotions (*M* = 98.33, *SD* = 1.37, *Mdn* = 98.00). Also, as shown in Table 4, for females, accuracy rates under high-arousal positive emotions (*M* = 98.00, *SD* = 1.55, *Mdn* = 98.00) and low-arousal positive emotions (*M* = 99.17, *SD* = 0.98, *Mdn* = 99.50) show no significant difference (*Z* = 1.63, *p* = 0.100). In comparison, for reaction time, males exhibit significantly shorter reaction times (*Z* = 2.20, *p* = 0.028, *Cohen*′*sd* = 1.16) under high-arousal negative emotions (*M* = 26449.17, *SD* = 4046.69, *Mdn* = 26046.00) compared to high-arousal positive emotions (*M* = 27511.17, *SD* = 3594.44, *Mdn* = 30373.00). For females, reaction times are significantly shorter (*Z* = 1.99, *p* = 0.046, *Cohen*′*sd* = 0.79) under high-arousal positive emotions (*M* = 30362.50, *SD* = 5326.55) compared to low-arousal positive emotions (*M* = 33004.17, *SD* = 8145.73).

**TABLE 3:** Influence of emotion on AX-CPT task performance for males.

Emotion dimension	Reaction time (M ± SD)	Accuracy (M ± SD)
Positive-valence, high-arousal	29183.00 ± 4295.00	98.33 ± 1.37
Positive-valence, low-arousal	27844.00 ± 5833.00	97.83 ± 1.72
Negative-valence, high-arousal	26449.00 ± 4047.00	97.67 ± 1.63
Negative-valence, low-arousal	27511.00 ± 3594.00	98.17 ± 1.33

**TABLE 4:** Influence of emotion on AX-CPT task performance for females.

Emotion dimension	Reaction time (M ± SD)	Accuracy (M ± SD)
Positive-valence, high-arousal	30362.50 ± 5326.55	98.00 ± 1.55
Positive-valence, low-arousal	33004.17 ± 8145.73	99.17 ± 0.98
Negative-valence, high-arousal	30251.67 ± 6598.45	99.17 ± 1.17
Negative-valence, low-arousal	29648.67 ± 6490.87	99.00 ± 1.10

There results suggest that emotional valence and arousal do not significantly affect task accuracy performance across different genders, but significantly impact reaction time performance. Males display shorter reaction times, i.e., better sustained attention, under high-arousal negative emotions than high-arousal positive emotions, while females display shorter reaction times under high-arousal positive emotions than low-arousal positive emotions. These results correspond with previous studies such as Bradley et al. (2001), that found males to show greater physiological reactivity toward negative emotions, and females to show greater reactivity toward positive emotions.

### 3.3 Emotion and flow experience data

Flow experience scores are displayed in Table 5. The Wilcoxon signed-rank test is used to assess differences in flow experience across the four sessions of emotional dimensions. Results indicate that emotional valence (positive: *M* = 52.46, *SD* = 9.17, *Mdn* = 50.75 and negative: *M* = 55.71, *SD* = 9.41, *Mdn* = 57.50) and arousal (high: *M* = 54.58, *SD* = 8.68, *Mdn* = 55.25 and low: *M* = 53.58, *SD* = 9.34, *Mdn* = 52.25) do not pose significant impact on flow experience during the AX-CPT (valence: *Z* = 1.49, *p* = 0.136 and arousal: *Z* = 0.45, *p* = 0.656). When accounting for gender, no significant difference (*Z* = 0.32, *p* = 0.750) in flow experience is found for males between high-arousal negative emotions (*M* = 50.67, *SD* = 10.29, *Mdn* = 50.50) and high-arousal positive emotions (*M* = 51.50, *SD* = 3.67, *Mdn* = 50.00). Neither is significant differences (*Z* = 0.68, *p* = 0.500) found for females between high-arousal positive emotion (*M* = 54.67, *SD* = 12.45, *Mdn* = 54.50) and low-arousal positive emotion (*M* = 56.00, *SD* = 11.35, *Mdn* = 56.00) conditions.

**TABLE 5:** Influence of emotion on flow experience.

Emotion dimension	Flow experience score (M ± SD)
Positive-valence	52.46 ± 9.17
Negative-valence	55.71 ± 9.41
High-arousal	54.58 ± 8.68
Low-arousal	53.58 ± 9.33

### 3.4 EEG data analysis

Differential brain activity due to variations in attention are commonly reflected in the frequency bands (Wang et al., 2011). Here, we extract and compare frequency domain characteristics, specifically Power Spectral Density (PSD), to reflect the changes of sustained attention in relation to emotion. PSD describes the distribution of signal power across frequencies. We calculate the PSD of α, β, and γ bands, as well as the sustained attention formula $\frac{\beta }{\alpha +\theta }$, using the Welch method from the Python-MNE toolkit (Gramfort et al., 2013). In detail, the Welch method divides the signal into *n* segments that allow overlap, which improves the signal’s variance properties (Solomon, 1991), windows the data, and computes the average PSD of multiple segments. In particular, the Hanning window is chosen for windowing to mitigate spectral distortion caused by rectangular windows (Harris, 1978). Additionally, baseline correction is performed by subtracting PSD values from the baseline phase, i.e. the first 5 min of each session, from the PSD values of each session. Finally, these PSD values are averaged over α (7–13 Hz), β (14–29 Hz), and γ (30–47 Hz) bands, to be used for analysis.

**FIGURE 4:**
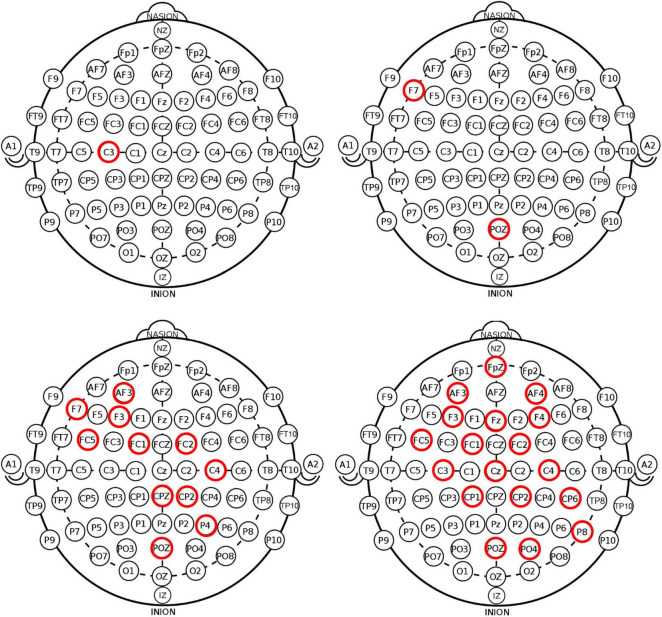
Patterns in EEG data that could represent sustained attention, for EEG power in α, β, γ bands, and $\frac{\beta }{\left(\alpha +\theta \right)}$ ratio.

For the sake of analysis, high and low sustained attention emotion conditions are divided based on AX-CPT task performance, where only the groups with significant differences are retained, resulting in 12 samples with high sustained attention and 12 samples with low sustained attention. Meanwhile, normality tests are conducted for EEG power in α, β, γ bands, and $\frac{\beta }{\alpha +\theta }$ ratio, for all subjects in each channel. The results suggest that the data does not have normality. Therefore, we use the Mann-Whitney U test to compare power differences between different sustained attention levels. We found the following patterns in EEG data that could represent sustained attention during the task, as shown in Figure 4, where the red circle represents the channels with significant differences between groups.

As shown in Table 6 and Figure 4a, the analysis of α band indicates that power in the C3 channel is significantly lower in high sustained attention state compared to low sustained attention state. This concurs with prior research, that found α wave activity in the C3 to be generally implicated in relaxation (Klimesch, 1999), meaning that decrease in activity can indicate heightened attention.

**TABLE 6:** Channels with significant differences in α band power by attention state.

Freq.	Channel	Sample size	High		Low		U	*p*	Cohen’s *d*
			Mean	SD	Mean	SD			
α	C3	24	0.0093	0.0017	0.0107	0.0140	38.0	0.050*	0.14

*p < 0.05.

As shown in Table 7 and Figure 4b, for β band, analysis indicate that the power in the F7 and POZ channels is significantly higher in high sustained attention state compared to low sustained attention state. Correspondingly, β wave activity has been associated with attention and alertness (Rouhinen et al., 2013).

**TABLE 7:** Channels with significant differences in β band power by attention state.

Freq.	Channel	Sample size	High		Low		U	*p*	Cohen’s *d*
			Mean	SD	Mean	SD			
β	F7	24	0.0068	0.0029	0.0045	0.0004	34.0	0.027*	0.66
	POZ	24	0.0070	0.0017	0.0060	0.0049	37.5	0.046*	0.27

*p < 0.05.

As shown in Table 8 and Figure 4c, for γ band, the analysis show that powers in the AF3, C4, CP2, CZ, F3, F7, FC1, FC2, FC5, P4, and POZ channels are significantly higher in high sustained attention state compared to low sustained attention state. In congruence with prior studies, γ wave activity is closely related to higher cognitive functions and information processing (Masuda, 2009; Rouhinen et al., 2013), which may be more active during high sustained attention.

**TABLE 8:** Channels with significant differences in γ band power by attention state.

Freq.	Channel	Sample size	High		Low		U	*P*	Cohen’s *d*
			Mean	SD	Mean	SD			
γ	AF3	24	0.0044	0.0011	0.0034	0.0005	33.0	0.023*	1.17
	C4	24	0.0045	0.0011	0.0032	0.0006	36.5	0.040*	1.47
	CP2	24	0.0046	0.0009	0.0038	0.0007	37.5	0.045*	0.99
	CZ	24	0.0044	0.0009	0.0037	0.0006	36.0	0.037*	0.92
	F3	24	0.0043	0.0010	0.0035	0.0005	36.0	0.037*	1.01
	F7	24	0.0056	0.0028	0.0031	0.0006	26.5	0.007**	1.23
	FC1	24	0.0044	0.0009	0.0036	0.0006	35.0	0.031*	1.05
	FC2	24	0.0044	0.0010	0.0036	0.0006	35.5	0.017*	0.97
	FC5	24	0.0045	0.0014	0.0034	0.0007	36.5	0.040*	0.99
	P4	24	0.0052	0.0011	0.0043	0.0012	36.5	0.020*	0.78
	POZ	24	0.0053	0.0016	0.0040	0.0008	34.0	0.027*	1.03

*p < 0.05,

**p < 0.01.

For $\frac{\beta }{\alpha +\theta }$ ratio, the analysis indicates that this ratio is significantly higher in high sustained attention state compared to low sustained attention state, as shown in Table 9 and Figure 4d. This is consistent with research, that suggested the $\frac{\beta }{\alpha +\theta }$ ratio reflects changes in attention and alertness (Wang et al., 2011).

**TABLE 9:** Channels with significant differences in $\frac{\beta }{\alpha +\theta }$ ratio by attention state.

Freq.	Channel	Sample size	High		Low		U	*p*	Cohen’s *d*
			Mean	SD	Mean	SD			
$\frac{\beta }{\alpha +\theta }$	AF3	24	0.2943	0.0260	0.2675	0.0315	32.0	0.021*	0.93
	AF4	24	0.3206	0.0493	0.2760	0.0259	28.0	0.010*	1.13
	C3	24	0.3018	0.0190	0.2712	0.0377	27.0	0.008**	1.03
	C4	24	0.3021	0.0291	0.2665	0.0298	28.0	0.010*	1.21
	CP1	24	0.2995	0.0158	0.2687	0.0398	36.0	0.039*	1.02
	CP2	24	0.2958	0.0186	0.2634	0.0336	31.0	0.017*	1.19
	CP6	24	0.3300	0.0315	0.2943	0.0321	31.0	0.017*	1.12
	CZ	24	0.2814	0.0214	0.2565	0.0279	37.0	0.045*	0.99
	F3	24	0.2878	0.0214	0.2645	0.0323	28.0	0.010*	0.85
	F4	24	0.2973	0.0455	0.2570	0.0266	32.0	0.020*	1.08
	FC1	24	0.2792	0.0188	0.2573	0.0257	36.0	0.039*	0.97
	FC2	24	0.2810	0.0296	0.2537	0.0278	37.0	0.045*	0.95
	FC5	24	0.3039	0.0391	0.2738	0.0499	35.0	0.033*	0.67
	FPZ	24	0.3424	0.0921	0.2704	0.0250	25.0	0.003**	1.07
	FZ	24	0.2755	0.0251	0.2943	0.0321	37.0	0.043*	0.65
	P4	24	0.3249	0.0271	0.2923	0.0482	35.0	0.033*	0.83
	P8	24	0.3760	0.0429	0.3316	0.0400	29.0	0.012*	1.07
	POZ	24	0.3126	0.0476	0.2581	0.0344	23.0	0.004**	1.31
	PO4	24	0.3255	0.0171	0.2996	0.0400	36.0	0.039*	0.84

*p < 0.05,

**p < 0.01.

EEG results from this study indicate significant differences between high and low sustained attention conditions in the power of α, β, γ bands, as well as $\frac{\beta }{\alpha +\theta }$ ratio. In high sustained attention state, EEG power in α band decreases in the C3 channel, β band power increases in the F7 and POZ channels, and both γ band power and $\frac{\beta }{\alpha +\theta }$ increase in the parietal and frontal regions. These findings corroborate previous studies that found α wave power decrease, as well as β and γ wave power increase during high sustained attention states. This might be due to the increase in cognitive resources required for high sustained attention tasks (Başar et al., 2001; Klimesch, 1999).

### 3.5 PPG data analysis

We also examine heart rate variability (HRV) characteristics in participants under high and low sustained attention states, calculated from PPG data. Considering the sample size in general PPG data analysis, we divided each task into two halves, and HRV features are extracted from each half, resulting in 48 samples. After normality testing, HRV indices VLF[%], LF[%], HF[%], LF/HF, LF[n.u.], and HF[n.u.] follow a normal distribution, while MEANRR, MEDIANRR, MEANHR, SDNN, RMSSD, NN50, pNN50, VLF[abs], LF[abs], and HF[abs] do not.

Indices that adhere to normality are analyzed with *t*-test. Among these, HF[%] shows a significant decrease in high sustained attention state (*M* = 35.94, *SD* = 10.03) compared to low sustained attention state (*M* = 42.24, *SD* = 10.80), *t*(46)=2.09, *p* = 0.042, *Cohen*′*sd* = 0.60. Indices that do not adhere to normality are analyzed with the Mann-Whitney U test. Among them, VLF[abs] displays a significant increase in high sustained attention state (*Md* = 868.66) compared to low sustained attention state (*Md* = 500.26), *U* = 176.50, *p* = 0.021, *Cohen*′*sd* = 0.82. These findings align with previous research that found high sustained attention tasks to require more cognitive resources, which lead to changes in autonomic nervous system regulation: reduced parasympathetic activity, associated with decrease in HF[%], and increased sympathetic activity, associated with increase in VLF[abs] (Krygier et al., 2013; Thayer et al., 2012).

## 4 Discussion

As elaborated in previous sections, literature on emotions’ effect on attention has yielded mixed results. This study takes a step toward resolving existing contradictions by improving upon methodology: using more ecologically valid VR videos to induce emotions, and measuring sustained attention directly with EEG and PPG. Additionally, the study enhances the analysis by taking gender differences into consideration and using sustained attention as a factor to account for the quality of attention. Results show that for females, sustained attention levels (i.e. quality of attention) are significantly higher during high-arousal positive emotions compared to low-arousal positive emotions, while for males, sustained attention levels during high-arousal negative emotions are significantly higher than during high-arousal positive emotions. In particular, the findings of this study could be applied to educational settings to enhance learning outcomes. For example, understanding the impact of emotions on sustained attention could inform instructional design, suggesting that educators might tailor learning environments to evoke positive high-arousal states in students, potentially improving their engagement and performance. Similarly, in professional training and workplace settings, creating emotionally positive and stimulating environments could enhance employees’ focus and productivity.

Moreover, this study clarifies the relationship between flow experience and sustained attention, showing that there is no significant association under the context of timed AX-CPT tasks. This corresponds with previous research testing flow experience with timed tasks, such as Ullen et al. (2012), but points toward a possible link between flow experience and ecological validity of the experiment task. At the same time, our analysis on EEG and PPG provide insight into how heightened sustained attention is directly reflected in brain activity. Results from EEG data enables looking specifically at frequency bands related to attention and sustained attention, while raising a concern that may be related to the mixed results in previous research. That is, in subsequent studies, it may be worth considering using similar direct measurements to further distinguish between responses resulting from high sustained attention versus from emotional arousal.

This study uses a relatively small sample size. Future studies in related directions should consider using larger samples, while taking gender differences and quality of sustained attention into account when analyzing attention task performance. Potential future research directions can include exploring the application of these results to other types of attention tasks with more ecological validity, such as reading, writing, gaming, as well as untimed tasks. Also, researchers could investigate the adaptive contexts that brought forth these gender differences in emotion induction responses. In doing so, the effects of emotion on attention/engagement and flow experience in different contexts could be further explored, to point toward a more systematic, unified theory, that could be applied to improve performance in complex real-world contexts. This broader application could guide the development of more effective strategies in education, training, and therapy, ultimately enhancing individual performance and well-being.

## Data Availability

The raw data supporting the conclusions of this article will be made available by the authors, without undue reservation.
